# Long-term outcomes of image-guided ablation and laparoscopic partial nephrectomy for T1 renal cell carcinoma

**DOI:** 10.1007/s00330-022-08719-1

**Published:** 2022-04-06

**Authors:** Vinson Wai-Shun Chan, Filzah Hanis Osman, Jon Cartledge, Walter Gregory, Michael Kimuli, Naveen S. Vasudev, Christy Ralph, Satinder Jagdev, Selina Bhattarai, Jonathan Smith, James Lenton, Tze Min Wah

**Affiliations:** 1 School of Medicine, Faculty of Medicine and Health, University of Leeds, Leeds, England UK; 2 Department of Urology, St. James’s University Hospital, Leeds, UK; 3 Leeds Institute of Clinical Trials Research, University of Leeds, Leeds, UK; 4 Department of Medical Oncology, St. James’s University Hospital, Leeds, UK; 5 Department of Pathology, St. James’s University Hospital, Leeds, UK; 6 Department of Diagnostic and Interventional Radiology, Institute of Oncology, Leeds Teaching Hospitals Trust, St. James’s University Hospital, Leeds, England LS9 7TF UK

**Keywords:** Cryoablation, Image-guided ablation, Partial nephrectomy, Radio-frequency ablation, Renal cell carcinoma

## Abstract

**Objective:**

To compare long-term outcomes and peri-operative outcomes of image-guided ablation (IGA) and laparoscopic partial nephrectomy (LPN).

**Material and methods:**

This is a retrospective cohort study of localised RCC (T1a/bN0M0) patients undergoing cryoablation (CRYO), radio-frequency ablation (RFA), or LPN at our institution from 2003 to 2016. Oncological outcomes were compared using Cox regression and log-rank analysis. eGFR changes were compared using Kruskal-Wallis and Wilcoxon-rank tests.

**Results:**

A total of 296 (238 T1a, 58 T1b) consecutive patients were identified; 103, 100, and 93 patients underwent CRYO, RFA, and LPN, respectively. Median follow-up time was 75, 98, and 71 months, respectively. On univariate analysis, all oncological outcomes were comparable amongst CRYO, RFA, and LPN (*p* > 0.05). On multivariate analysis, T1a patients undergoing RFA had improved local recurrence-free survival (LRFS) (HR 0.002, 95% CI 0.00–0.11, *p* = 0.003) and metastasis-free survival (HR 0.002, 95% CI 0.00–0.52, *p* = 0.029) compared to LPN. In T1a and T1b patients combined, both CRYO (HR 0.07, 95% CI 0.01–0.73, *p* = 0.026) and RFA (HR 0.04, 95% CI 0.03–0.48, *p* = 0.011) had improved LRFS rates. Patients undergoing CRYO and RFA had a significantly smaller median decrease in eGFR post-operatively compared to LPN (T1a: *p* < 0.001; T1b: *p* = 0.047). Limitations include retrospective design and limited statistical power.

**Conclusions:**

IGA is potentially as good as LPN in oncological durability. IGA preserves kidney function significantly better than LPN. More studies with larger sample size should be performed to establish IGA as a first-line treatment alongside LPN.

**Key Points:**

• *Ablative therapies are alternatives to partial nephrectomy for managing small renal cell carcinomas.*

• *This study reports long-term outcomes of image-guided ablation versus partial nephrectomy.*

• *Ablative therapies have comparable oncological durability and better renal function preservation compared to partial nephrectomy.*

**Supplementary Information:**

The online version contains supplementary material available at 10.1007/s00330-022-08719-1.

## Introduction

Traditionally, patients with small renal masses (SRMs) are managed by radical or partial nephrectomies (PNs). However, laparoscopic PNs (LPNs) are associated with significant complication rates (~20%) [1]. Percutaneous image-guided radio-frequency ablation (RFA) in SRMs was first reported in 1997 [2]. The adoption of image-guided ablation (IGA) has rapidly increased in the management of SRMs due to its minimally invasive nature and the theoretical ability to offer preservation of renal function and lower complication rate when compared to PN [3]. Other energy sources have been adopted to manage SRMs, including cryoablation (CRYO) [4], microwave ablation [5], and, more recently, irreversible electroporation [6].

The current European Association of Urology (EAU) guidelines suggest strong evidence to perform PN for T1 renal masses, and weak evidence to only offer IGA to those with significantly co-morbidity and frailty [7]. The EAU guidelines have also suggested IGA to be associated with higher rates of recurence, although unlikely after 5 years, based on limited evidence [7]. On the other hand, the American Urological Association (AUA) guidelines suggest thermal ablation as an alternative approach in managing cT1a tumours; however, the lack of high-quality literature with long follow-up periods of patients with confirmed histology was emphasised [8]. The AUA guidelines also specify the importance of long follow-up periods (> 5 years) to accurately assess for late local recurrences. While there are some non-randomised evidence base to perform PN over radical nephrectomy, there is only one study by Andrews et al, showing comparable long-term oncological outcomes of IGA and LPN for SRMs for up to 5 years[3, 9]. Chang et al, had also shown comparable 5-year outcomes between laparoscopic or imaged-guided RFA and PN [10]. Furthermore, the overall quality of studies comparing IGA and LPN is limited. Single-arm studies have suggested effective long-term cancer control in patients undergoing percutaneous RFA at 10 years [11].

While there is a desperate need for a high-quality randomised controlled trial to compare RFA, CRYO, and LPN, prospective recruitment has proven to be difficult as seen by the SURAB feasilibity study and the CONSERVE trial, which both failed in recruitment [12, 13]. This study aims to provide 10 years of experience and evidence to inform guidelines for long-term oncological outcomes in patients undergoing image-guided CRYO or RFA and LPN for biopsy- or histology-proven T1aN0M0 and T1bN0M0 renal cell carcinomas (RCCs).

## Methods

### Study design

This is a retrospective analysis of a prospectively maintained registry from 2003 to 2016. Following institutional health and research authority approval, consecutive adult patients who underwent image-guided CRYO, RFA, or laparosocpic LPN for cT1N0M0 histology-confirmed RCC were included for the study. The patient selection process at our institution was previously described [14]. cT1 renal masses were defined as a maximum tumour diameter of ≤ 7 cm limited to the kidney on radiographic imaging according to the American Joint Committee on Cancer staging manual [15]; with cT1 further divided to cT1a (≤ 4 cm) and cT1b (> 4 cm and ≤ 7 cm). Patients presenting with multiple renal tumours, recurrence, inherited RCC syndromes, or a solitary kidney were excluded from the analysis [16]. Patients with a history of LPN, CRYO, or RFA of the same kidney were also excluded from analysis. Primary outcome of the study was to evaluate and compare the long-term local recurrence-free survival (LRFS) between CRYO, RFA, and LPN. Secondary outcomes include overall survival (OS), cancer-specific survival (CSS), metastasis-free survival (MFS), rate and severity of complications, and change in renal function peri-operatively. The detailed methods of the performance of IGA and LPN are outlined in the supplementary appendix.

### Patient follow-up

The follow-up protocol for IGA was previously described in detail [14]. All patients were followed at 1, 3, and 6 months after the procedure and annually onwards for a period of 10 years using MRI or CT. Local recurrence was defined as new area(s) of enhancement in the zone of ablation after at least one imaging study had shown complete lack of enhancement in the treated area. Metastatic disease was defined as extra-renal disease on imaging confirmed or suspicioned to have originated from the kidney. Cancer-specific death was defined as any deaths from RCC.

### Clinical features, variables, covariates, and data acquisition

Patient clinical features such as age, sex, treatment date, follow-up details, histopathological details, R.E.N.A.L. nephrometry score [17], co-morbidities (according to the Charlson Comorbidity Index [CCI][18]), procedure details, complications (according to the Clavien Dindo Classification [19]), and estimated glomerular filtration rate (eGFR; CKD-EPI [20]) were extracted from the prospectively maintained database. Utilising the National Health Service (NHS) patient records, the patients were followed for their living status and cause of death until 25th January 2021.

### Outcomes and data synthesis

Differences in baseline characteristics were evaluated using the chi-square test and the Kruskal-Wallis test. CSS, OS, LRFS, and MFS were evaluated from the time of treatment to the time of event using the Kaplan-Meier method. Ten-year survival rates and corresponding 95% confidence intervals (95% CI) were reported. The Cox proportional hazard regression model was utilised to evaluate survival in CRYO, RFA, and LPN patients, reporting as hazard ratios (HRs), 95% CI, and *p*-values. To allow evaluation of HRs when no events were observed in an arm, an event was artificially created at the latest follow-up for that arm. Complication rates and severity were evaluated using the chi-squared test and logistic regression. Changes in peri-operative renal function were evaluated using the Kruskal-Wallis test and the Wilcoxon matched pairs signed rank sum test. Propensity score matching [21, 22] was intended to be used to compare RFA and CRYO with LPN. However, the groups were so different for a number of the key matching variables that this approach became impractical, as detailed in the “Results” section, and in the supplementary appendix. In order to facilitate comparison of the two groups, we therefore used Cox’s proportional hazards model [23], including all the variables we had intended to use in the matching analysis (age, sex, laterality, CCI, R.E.N.A.L nephrometry score, lesion size, RCC type, grade, and t-stage), to adjust for imbalances in these variables between the various treatment groups. This is, of course, not a substitute for a randomised trial, and the results have to be interpreted with a degree of caution. Furthermore, there are relatively few events, creating sensitivity issues with the model results. However, given the difficulties in undertaking such a randomised trial, and the time that such a trial would take to complete, we decided that this approach provides an appropriate means of performing and interpreting inter-group comparisons. Amongst 10 patients with missing CCI or R.E.N.A.L. nephrometry score, median CCI or nephrometry score was imputed. Sensitivity analyses have shown identical results and hence all patients were included in the final analyses. All analyses are two-tailed at a significance level of 0.05. All statistical analyses were performed on STATA/MP 16.0 (StataCorp).

## Results

A total of 290 patients were included in the analysis. Supplementary figure 1 shows how these patients were selected for inclusion in the study.

### Oncological outcomes in T1a patients using univariate analysis

#### Baseline characteristics of T1a patients

A summary of the clinical and pathological characteristics of the 238 T1a patients included in the analysis is given in Table 1. RCC histology, Fuhrman grade, age, tumour size, R.E.N.A.L nephrometry score, baseline eGFR and CCI were found to be significantly different between the three groups. The median (IQR) follow-up time was 75.6 (66.8–86.5) months, 106.0 (61.2–135.1) months, and 72 (64.6–99.7) months in CRYO, RFA, and LPN patients, respectively.

**Table 1:** Baseline characteristics of T1a patients

Modality	Cryoablation (*n* = 72)		RFA (*n* = 87)		PN (*n* = 79)		
Variable	Frequency	%	Frequency	%	Frequency	%	*p*-value
Sex							
Male	42	58.3	59	67.8	52	65.8	*p* = 0.435
Female	30	41.7	28	32.2	27	34.2	
Laterality							
Left	29	40.3	39	44.8	32	40.5	*p* = 0.659
Right	43	59.7	48.0	55.2	46	58.2	
Horseshoe	0	0.0	0	0.0	1	1.3	
RCC type							
Conventional	45	62.5	71	81.6	49	62.0	***p* < 0.001**
Papillary	6	8.3	5	5.7	17	21.5	
Oesinophil	2	2.8	6	6.9	1	1.3	
Chromophobe	19	26.4	5	5.7	12	15.2	
Fuhrman grade							
Ungraded	13	18.1	10	11.5	5	6.3	***p* < 0.001**
1	17	23.6	21	24.1	5	6.3	
2	35	48.6	40	46.0	27	34.2	
3	6	8.3	14	16.1	38	48.1	
4	1	1.4	2	2.3	4	5.1	
	Median	IQR	Median	IQR	Median	IQR	*p*-value
Age	72	62–76	73	66–78	59	49–67	***p* < 0.001**
Tumour size (cm)	2.85	2.5–3.45	2.80	2.4–3.4	2.5	2.1–3.0	***p* = 0.011**
R.E.N.A.L nephrometry score	6	5–7	6	5–8	5	4–7	***p* = 0.002**
Baseline eGFR	77.88	60.9–87.8	89.02	71.2–104.4	91.31	75.3–101.9	***p* < 0.001**
Charlson Comorbidity Index	3	2–4.5	4	3–5	2	1–4	***p* < 0.001**

#### Event-specific outcomes

**Fig. 1:**
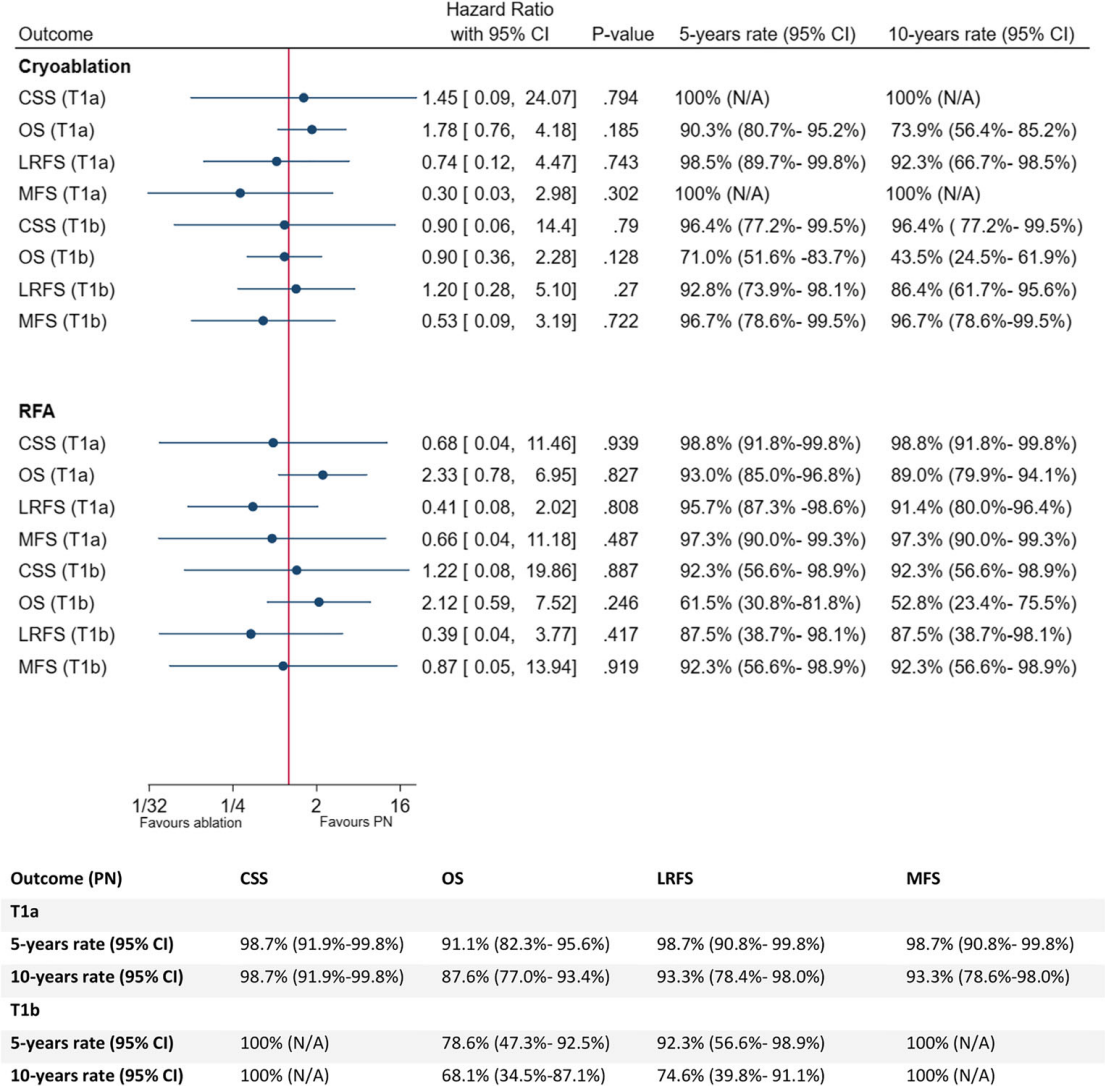
Forest plot summary of all oncological outcomes in T1a and T1b patients undergoing cryoablation or RFA compared to LPN using the univariate Cox proportional hazard model; 95% CI, 95% confidence interval; CSS, cancer-specific survival; OS, overall survival; LRFS, local recurrence-free survival; MFS, metastasis free-survival; RFA, radio-frequency ablation; LPN, partial nephrectomy

**Fig. 2:**
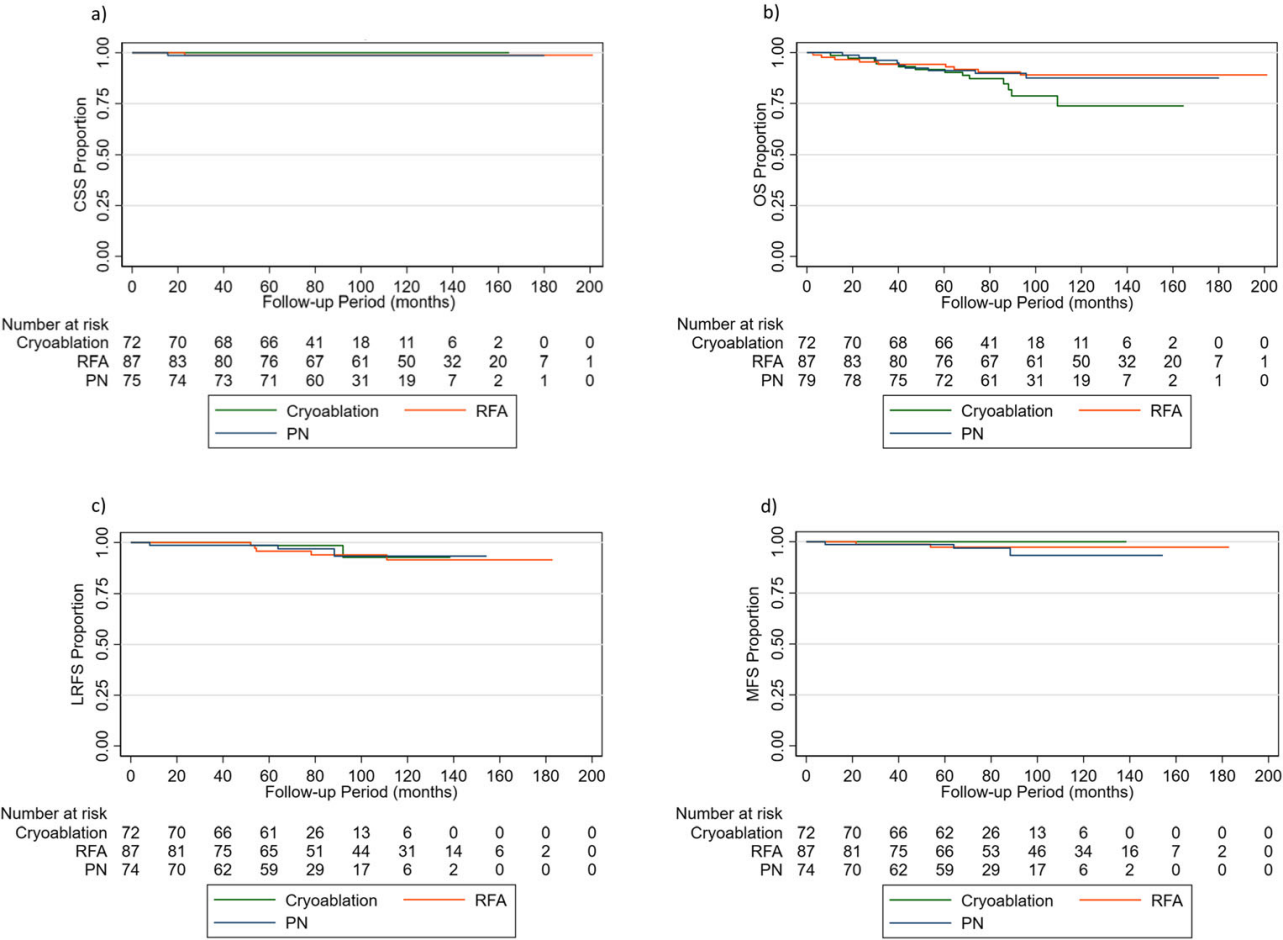
**a** Cancer-specific survival, (**b**) overall survival, (**c**) local recurrence-free survival, and (**d**) metastasis-free survival in T1a patients

Totals of 204, 238, 233, and 233 patients were evaluated for CSS, OS, LRFS, and MFS, respectively, with exclusions being for lack of follow-up (LRFS: 5, MFS: 5), and unknown causes of death (CSS: 4) in the LPN group only. Results were comparable between the 3 groups for all 4 endpoints (Figs. 1 and 2). Only two RCC-related deaths were observed: one in the RFA group and one in the LPN group. A total of 31 deaths were observed (CRYO: 13, RFA: 9, LPN: 9). Ten local recurrences were observed (CRYO: 2, RFA: 5, LPN: 3). Five metastatic events were observed (CRYO: 0, RFA: 2, LPN: 3). A total of 72 and 87 patients were evaluated for CRYO and RFA for all outcomes, respectively. A total of 75, 79, 74, and 74 patients undergoing LPN were evaluated for CSS, OS, LRFS, and MFS, respectively.

### Oncological outcomes in T1b patients on univariate analysis

**Fig. 3:**
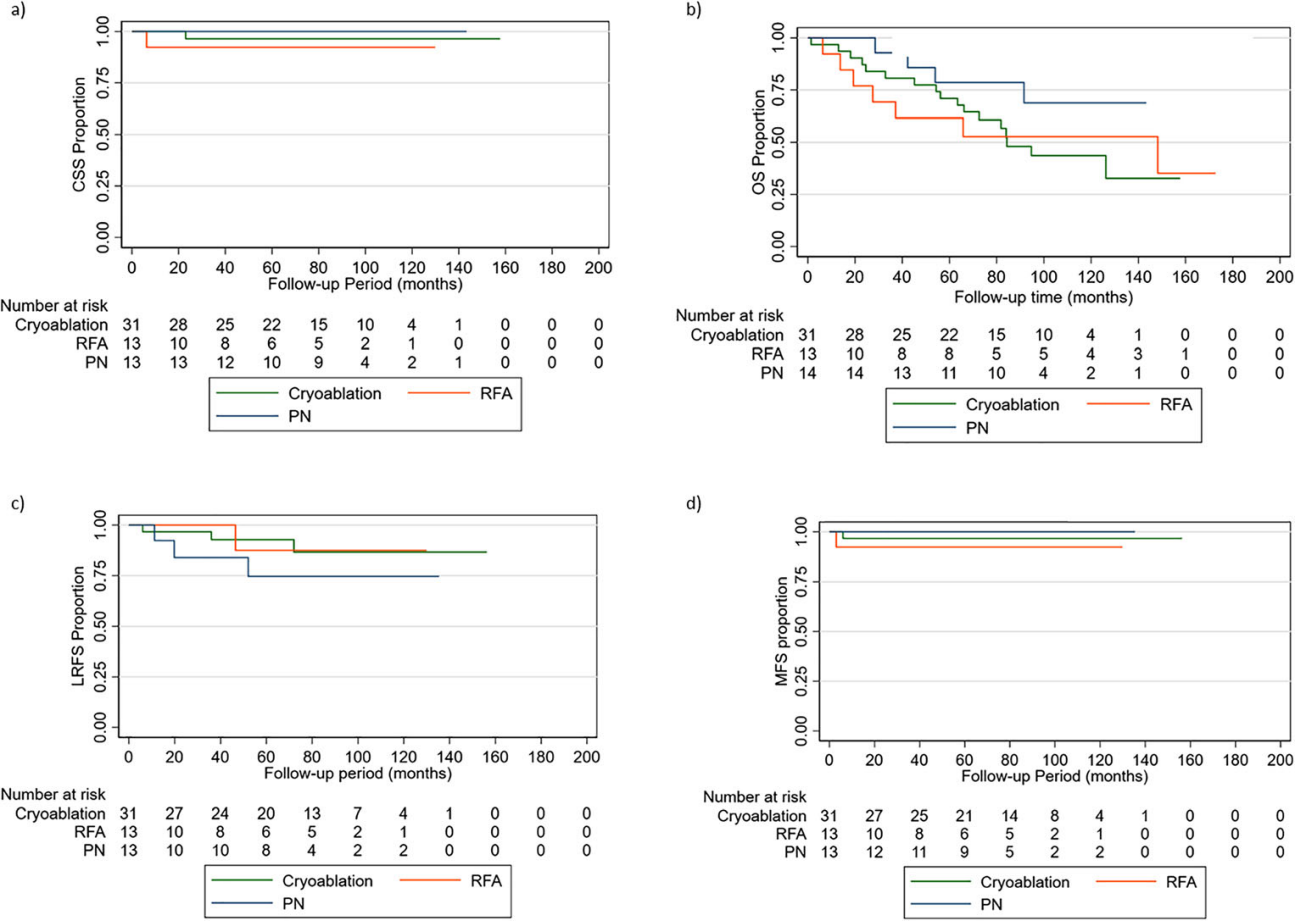
**a** Cancer-specific survival, **b** overall survival, **c** local recurrence-free survival, and **d** metastasis-free survival in T1b patients

A total of 58 T1b patients were included in this study. A summary of their clinical and pathological characteristics are outlined in Table 2. RCC histology, Fuhrman grade, age, tumour size, R.E.N.A.L nephrometry score, baseline eGFR, and CCI were found to be significantly different between the three groups. The median (IQR) follow-up duration is 72.5 (42.0–100.9) months, 59.5 (27.5–99.39) months, and 67.9 (50.8–91.3) months for CRYO, RFA, and LPN, respectively. CSS, OS, LRFS, and MFS are all comparable between patients undergoing CRYO, RFA, or LPN (Figs. 1 and 3). The details of the results are outlined in the supplementary appendix.

**Table 2:** Baseline characteristics of T1b patients

Modality	Cryoablation (*n* = 31)		RFA (*n* = 13)		PN (*n* = 14)		
Variable	Frequency	%	Frequency	%	Frequency	%	*p*-value
Sex							
Male	22	29.1	6	46.2	6	42.9	*p* = 0.897
Female	9	71.0	7	53.9	8	57.14	
Laterality							
Left	15	48.4	2	15.4	9	64.3	*p* = 0.656
Right	16	59.7	11	84.6	5	35.7	
Horseshoe	0	0.0	0	0.0	0	0	
RCC type							
Conventional	24	77.4	12	100.0	12	85.7	***p* < 0.001**
Papillary	2	6.5	0	0	1	7.1	
Oesinophil	0	0	0	0	1	7.1	
Chromophobe	5	16.1	0	0	0	0	
Fuhrman grade							
Ungraded	2	6.45	2	15.4	2	14.3	***p* < 0.001**
1	4	12.9	2	15.4	2	14.3	
2	19	61.3	6	46.2	2	14.3	
3	5	16.1	3	23.1	8	57.1	
4	1	3.2	0	0	0	0	
	Median	IQR	Median	IQR	Median	IQR	*p* - value
Age	77.0	65–80	78	65–79	57	44–67	***p* < 0.001**
Tumour size (cm)	4.5	4.10–5.10	4.5	4.5–4.8	4.45	4.2–5.3	***p* = 0.001**
R.E.N.A.L nephrometry score	9	7–10	7	7–9	7	5–8	***p* = 0.002**
Baseline eGFR	57.6	42.8– 79.2	37.3	30.5–43.4	84.8	73.3–97.1	***p* < 0.001**
Charlson Comorbidity Index	4	3–6	3	2.5–4.0	3	0.5–4.5	***p* < 0.001**

### Post-operative complications

The rate and severity of post-operative complications for all three modalities were found to be similar in both cT1a (CRYO: 11.1%, RFA: 18.4%, LPN: 14.1%) and cT1b patients (CRYO: 19.4%, RFA: 15.4%, LPN: 7.7%). Both logistic regression and multinomial logistic regression did not show significant difference between the three groups’ rate and severity of complications (Supplementary Table 1 and 2). A summary of all complications occurring during the study period are reported in Supplementary Table 3.

### Change in renal function

The post-operative eGFR and change in eGFR peri-operatively of T1a and T1b patients undergoing CRYO, RFA, and LPN are shown in Table 3. Only small changes in eGFR were found in patients undergoing CRYO and RFA, as compared to substantial falls in eGFR in LPN patients (Wilcoxon matched pairs signed rank sum Z and *p*-values; CRYO: 3.0, 0.003, RFA: 2.4, 0.02, LPN: 6.0, < .0001). When comparing the change in renal function peri-operatively using the Wilcoxon 2-sample rank sum test, in both T1a (Z = 4.1, *p* < .0001) and T1b (Z = 2.5, *p* = .01) patients, those undergoing IGA had a significantly smaller median change in eGFR compared to LPN (Table 3).

**Table 3:** Peri-operative change in eGFR in T1a and T1b patients undergoing image-guided cryoablation, RFA, and PN

Outcome	T-stage	Cryoablation	RFA	PN	*p*-value (Kruskal-Wallis test)
Pre-operative eGFR (ml/min/1.73 m^2^; median [IQR])	T1a	77.88 (60.9–87.8)	89.0 (71.2–104.4)	91.3 (75.3–101.9)	< 0.001
	T1b	57.6 (42.8–79.2)	37.3 (30.5–43.4)	84.8 (73.3–97.1)	< 0.001
Post-operative eGFR (ml/min/1.73 m^2^; median [IQR])	T1a	68.9 (52.9–85.7)	88.5 (70.6–100.82)	75.2 (60.4–92.0)	< 0.001
	T1b	56.4 (34.1–77.8)	40.0 (27.8–49.9)	61.5 (44.2–80.0)	0.078
Change in eGFR pre-and post-operatively (ml/min/1.73 m^2^; median [IQR])	T1a	−1.39 (−7.51–1.28)	−2.42 (−9.1–3.8)	−7.40 (−18.1 to −2.8)	< 0.001
	T1b	−2.11 (−7.6–1.1)	−1.52 (−2.7–2.2)	−13.3 (−39.9 to −1.1)	0.023
Percentage change in eGFR pre- and post-operatively (%; median [IQR])	T1a	−2.19 (−11–2.5)	−3.44 (−10.0–4.6)	−9.35 (−22.5 to −3.00)	< 0.001
	T1b	−5.05 (−15.6–1.4)	−1.70 (−9.4–5.9)	−24.6 (−41.9 to −11.3)	0.047

*RFA*, radio-frequency ablation; *PN*, partial nephrectomy; *eGFR*, estimated glomerular filtration rate; *IQR*, interquartile range

### Results of propensity-score matching and multivariate analysis

Initially, it was intended to explore the propensity score matching approach, as described in the “Methods” section. However, this proved to be infeasible due to large differences in baseline factors between the treatment groups, most substantially in age (Supplementary Figure 4; Tables 1 and 2). Further details, results, and explanation are given in supplementary Figures 2 and 3. Therefore, as described in the “Methods” section, the Cox multivariate method was used to adjust for these imbalances and compare the treatment arms (Table 4). As events are relatively scarce in this study, sensitivity analyses were performed by replacing an event with censoring at that time (results not presented). Minimal differences to the results presented were observed for all of the outcomes, demonstrating that the results are relatively insensitive to such small changes, and are therefore relatively robust. Certainly, the overall findings would be unchanged as a result of a single patient having a different outcome.

**Table 4:** Oncological outcomes in T1a and combined T1a/T1b patients in multivariate Cox proportional hazards model

Modality	Cryoablation		RFA		Cryoablation and RFA combined	
Outcome	HR (95% CI)	*p*-value	HR (95% CI)	*p*-value	HR (95% CI)	*p*-value
T1a only						
CSS	Not estimated*		Not estimated*		Not estimated*	
OS	1.30 (0.33–5.11)	0.708	0.70 (0.15–3.34)	0.657	0.66 (0.19–2.39)	0.544
LRFS	0.003 (0.00–2.39)	0.087	0.002 (0.00–0.11)	0.003	0.006 (0.00–0.15)	0.002
MFS	Not estimated*		0.002 (0.00–0.52)	0.029	0.002 (0.00–0.51)	0.028
T1a and T1b						
CSS	Not estimated*		Not estimated*		0.0001 (0.00–7876.7)	0.323
OS	1.20 (0.47–3.59)	0.613	0.62 (0.19–2.03)	0.426	0.73 (0.30–1.77)	0.487
LRFS	0.07 (0.01–0.73)	0.026	0.04 (0.03–0.48)	0.011	0.08 (0.01–0.44)	0.004
MFS	Not estimated*		0.19 (0.01–3.10)	0.242	0.13 (0.01–2.22)	0.158

*RFA*, radio-frequency ablation; *HR*, hazard ratio; *CSS*, cancer-specific survival; *OS*, overall survival; *LRFS*, local recurrence-free survival; *MFS*, metastasis-free survival

*Not estimated due to the limited number of events

In univariate Kaplan-Meier analyses, IGA and LPN were shown to have comparable LRFS. However, given that the CRYO and RFA groups consist of patients with considerably worse prognostic factors, after multivariate adjustment, CRYO and RFA appear to be superior to LPN for LRFS. The magnitude of the effect in the two ablative therapy groups is almost identical (see Supplementary Figure 5) so a combined group analysis, stratified by group, was performed, demonstrating ablative therapies to be superior to LPN for LRFS (HR 0.006, 95% CI 0.00–0.15, *p* = 0.002). Note that the RFA/LPN comparison reaches statistical significance on its own (Table 4; *p* = 0.003), and, although the CRYO/LPN result is not statistically significant (Table 4, *p* = 0.087), this is largely a result of paucity of patient and event numbers. Although effect sizes (HR) appear to be substantial for statistically significant outcomes (LRFS, MFS), suggesting extreme advantage to IGA patients, they are unlikely to reflect real effect sizes due to a combination of the extreme selection bias. Finally, the lower 90% confidence interval on the hazard ratio is less than 1 for CRYO (Supplementary Table 5), which demonstrates at least 90% confidence that CRYO is as good as LPN for LRFS. For clarity, characteristics of all patients with T1a tumours and subsequent local recurrences are shown in Table 5.

**Table 5:** Characteristics of all patients with T1a tumour and local recurrences

Intervention	Age	Sex	Laterality	CCI	Pre-operative eGFR	Tumour grade	R.E.N.A.L. nephrometry score	Lesion size	RCC type	LRFS duration (months)	Outcome
Cryoablation	74	Male	Right	6	56.7	2	8	3.6	Clear cell	52	Dead unrelated to RCC
Cryoablation	76	Male	Right	3	79.5	2	7	2.7	Chromophobe	76	Alive
(Cryoablation mean)	75			4.5	68.07	2	7.5	3.15		64	
RFA	68	Male	Right	4	103.2	3	8	3.3	Clear cell	54	Alive
RFA	78	Female	Left	3	78.4	3	9	3.5	Clear cell	54	Alive
RFA	77	Female	Right	3	52.2	3	7	2.7	Clear cell	111	Alive
RFA	83	Male	Right	6	121.5	1	9	3.3	Clear cell	52	Alive
RFA	73	Female	Left	4	75.6	3	8	3	Clear cell	78	Alive
(RFA mean)	75.8			4	86.2	2	8.2	3.16		69.8	
PN	65	Female	Left	2	79.7	3	9	1.8	Clear cell	64	Alive
PN	64	Male	Left	4	74.5	3	4	3.4	Clear cell	88	Alive
PN	65	Male	Right	3	93.3	3	7	3.4	Clear cell	8	Dead from RCC
(PN mean)	64.7			3	82.5	3	6.7	2.87		53.3	
Overall mean	72.3			3.8	81.4	2.3	7.6	3.07		62.4	

*CCI*, Charlson Comorbidity Index; *eGFR*, estimated glomerular filtration rate; *RCC*, renal cell carcinoma; *LRFS*, local recurrence-free survival; *RFA*, radio-frequency ablation; *PN*, partial nephrectomy

## Discussion

The number of high-quality studies comparing the use of IGA and LPN is scarce, with most limited by extreme selection bias and short follow-up periods [3, 24].

The univariate analysis results hereby reported are similar to that reported by Andrews et al [9] in 2019 and a recent published meta-analysis [3] as CSS, LRFS, and MFS were found to be comparable amongst the three modalities in both T1a and T1b patients. However, the available studies only assessed outcomes up to 5 years. In this cohort, as a result of serious selection bias, where LPN patients are signifcantly younger and less comorbid and have smaller tumours, propensity score matching was impossible. Therefore, a multivariate Cox proportional hazards model approach was utilitsed. In the multivariate analysis, we have found all oncological outcomes are at least comparable. Although LRFS is shown to be superior in T1a and T1b patients undergoing RFA (*p* = 0.011) and CRYO (*p* = 0.026), given the small number of events, model sensivity issues, and the fact that this is not a randomised trial, it is perhaps inappropriate to think that the results demonstrate superiority for ablative therapies. However, it seems reasonable to conclude that IGAs are at least as good as the surgical alternative. Furthermore, in contrast to the EAU’s guidance [7], our results have shown that recurrences after 5 years may have been more common than usually perceived, with five recurrences observed after 5 years (Table 5).

Despite selection bias, in contradiction to previous cohorts [9, 25] and a recent meta-analysis [3], our study did not find OS to be significantly different in the three treatment arms in both T1a and T1b patients. Andrews et al have reported 5-year OS to be significantly worse in CRYO and RFA patients with T1a/T1b disease even after propensity matching and subgroup analysis in patients with RCC [9]. The positive finding in our study could be the result of the extended follow-up time, offsetting potential selection bias arising over age of the included patients. Furthermore, life expectancy in the UK is significantly higher than that in the USA, further offsetting the age selection bias in the study [26]. While age is commonly regarded as a confounder in similar studies, our study found it be a significant, but only small predictor of overall survival in patients with T1a tumours in this cohort (HR 1.05, 95% CI 1.01–1.08, *p* = 0.016), explaining the minimal effect of selection bias on our results.

The rate and severity of complications in our study was not significantly different amongst the three modalities. This is in line with recent studies and with the meta-analysis of percutaneous IGA and PN [3]. While no theoretical advantage of reduced complications is observed in the literature, the learning curve for both LPN and percutaneous IGA is at about 100 cases [14, 27, 28], and few results have been reported for centres significantly beyond the learning curve [3].

As expected, as renal parachyma is better preserved in CRYO and RFA, our study found little or no change in eGFR in patients undergoing CRYO and RFA, as compared to a significant fall of eGFR in LPN patients. Although not investigated in this study, this will help inform treatment decisions in those with solitary kidneys or impaired renal function.

The strengths of this study include long-term follow-up, inclusion of R.E.N.A.L nephrometry scores and confirmed RCC status. While the results may be positive, this study does not come without its limitations. Firstly, our sample size (especially with T1b patients) is too small to be well-powered statistically. Secondly, the study is also limited by strong selection bias owing to the retrospective study design. This is evident by the inability to perform propensity score matching, with attempted mitigation using multivariate analysis. However, despite the seletion bias, the results are still positive. Thirdly, it is recognised that treatment options may depend on the location of the tumour and the nephrometry score may not be a complete representation of tumour complexity for treatment. Ultimately, it may not be entrirely safe to treat central tumours with CRYO, RFA, or even LPN, and radical nephrectomy may remain an option for some of the patients. Finally, the inclusion of only LPN may not be representative of patients undergoing robotic PN or open PN, as complication profiles and oncological outcomes may significantly differ [29].

The optimal investigations and management of small RCCs are debated, and there are factors that must be taken into consideration in order to compile evidence to allow better patient care. For example, the use of renal tumour biopsies should be considered, at least in a research context, as this allows evaluation of the treatment effects of malignant lesions without the biases arising from a proportion of benign results [30, 31] The use of active surveillance to manage small RCCs is becoming increasingly popular [32, 33], and the use of renal tumour biopsy prior to both active surveillance and IGA will allow for better comparison between the different managements of small RCCs.

This study reported long-term outcomes of patients undergoing CRYO, RFA, or LPN for T1a/T1b RCC. Although, in this cohort, patients undergoing CRYO and RFA have superior LRFS and comparable oncological outcome in general, the extreme selection bias and lack of events suggest the cautious conclusion that CRYO and RFA are at least as good as LPN in oncological outcomes. However, this study can conclude that CRYO and RFA have better renal function preservation compared to LPN. Therefore, percutaneous IGA, CRYO, and RFA should be potentially reflected in guidelines to be considered first-line treatment along with LPN for small RCCs providing more promising outcomes from larger prospective and multicentre cohorts can be made avaliable evaluating both the peri-operative and long-term outcomes for both T1a and T1b RCCs. The highly anticipated NEST trial [34], a RCT comparing LPN and IGA in T1a is designed to address the much needed level one evidence in this area.

## Supplementary information

ESM 1 (DOCX 153 kb)

## Abbreviations

(L)PN: (Laparoscopic) Partial Nephrectomy
AUA: American Urological Association
CCI: Charlson comorbidity index
CRYO: Cryoablation
CSS: Cancer-specific survival
EAU: European Association of Urology
IGA: Image-guided ablation
LRFS: Local recurrence-free survival
MFS: Metastasis-free survival
OS: Overall survival
RCC: Renal cell carcinoma
RFA: Radio-frequency ablation
SRM: Small renal mass

## Declarations

### Guarantor

The scientific guarantor of this publication is Vinson Wai-Shun Chan and Tze Min Wah.

### Statistics and biometry

Prof. Walter Gregory kindly provided statistical advice for this manuscript, he has significant statistical expertise.

### Methodology

• Retrospective

• Observational

• Performed at one institution
