# Supplementary material for: Long-term outcomes of image-guided ablation and laparoscopic partial nephrectomy for T1 renal cell carcinoma
Source: Eur Radiol. 2022 Apr 6;32(9):5811–20. doi: 10.1007/s00330-022-08719-1 (PMC9381474; doi:10.1007/s00330-022-08719-1)
Supplement: Supplementary file 1 — (DOCX 153 kb) [file 330_2022_8719_MOESM1_ESM.docx]

**Supplementary Material to “Long-term outcomes of image-guided ablation and laparoscopic partial nephrectomy for T1 renal cell carcinomas”**

**Authors: Vinson Wai-Shun Chan, Filzah Hanis Osman, Jon Cartledge, Walter Gregory, Michael Kimuli, Naveen Vasudev, Christy Ralph, Satinder Jagdev, Selina Bhattarai, Jonathan Smith, James Lenton, Tze Min Wah**

Table of Contents

[1. Technical details of RFA, Cryoablation and Partial Nephrectomy 3](#_Toc96877091)

[1.1. RFA and Cryoablation 3](#_Toc96877092)

[1.2. Partial Nephrectomy 3](#_Toc96877093)

[2. Supplementary Figure 1 – Patient selection process 4](#_Toc96877094)

[3. Supplementary Results to T1b subgroups 5](#_Toc96877095)

[4. Supplementary Table 1 - Logistic regression of post-operative complication rate 6](#_Toc96877096)

[5. Supplementary Table 2 - Multinomial logistic regression of severity of post-operative complications 7](#_Toc96877097)

[6. Supplementary Table 3 - Summary of all post-operative complications 8](#_Toc96877098)

[7. Results from attempted propensity score matching analysis 10](#_Toc96877099)

[Supplementary Figure 2 – Propensity score matching histogram of T1a RFA and PN patients 10](#_Toc96877100)

[Supplementary Figure 3 – Propensity score matching histogram of T1a Cryoablation and PN patients 11](#_Toc96877101)

[Supplementary Figure 4 - Distribution of age amongst partial nephrectomy, cryoablation and RFA; LPN: Partial Nephrectomy; RFA: Radio-frequency ablation; CRYO: Cryoablation 12](#_Toc96877102)

[8. Results of multivariate analysis 13](#_Toc96877104)

[Supplementary Figure 5 – Forest plot showing magnitude of effect in T1a patients undergoing cryoablation and RFA compared to PN. 13](#_Toc96877105)

[Supplementary Table 4 – Multivariate proportional hazards regression of local recurrence-free survival in T1a cryoablation or PN patients showing 90% confidence intervals for the estimated Hazards ratios. 14](#_Toc96877106)

# 1. Technical details of RFA, Cryoablation and Partial Nephrectomy

## 1.1. RFA and Cryoablation

Imaged-guided RFA was performed as described previously[15]. In essence, RFA was performed with varying sizes (3, 3.5 or 4 cm) of umbrella shaped multi-tines LeVeen CoAccess RFA needle electrode selected to match the size of the tumour. Pulsed RF currents were delivered by an impedance-controlled 200-W generator (Boston Scientific, MA, USA) under CT guidance.  For image-guided cryoablation, depending on the size and geometry of the tumour, between 4-8 cryoprobes were inserted into the tumour with two cycles of freezing-thawing treatment under CT guidance[17]. The ice-ball was formed at the tip of the cryoprobes to cover the whole tumour using the Joule-Thomson effect with Argon-Helium gas delivery from the cryoablation generator (Boston Scientific, MA, USA). All biopsies were performed under image-guidance at the time of ablation or as a separate session prior to ablation.

## 1.2. Partial Nephrectomy

All PNs were performed laparoscopically under general anaesthesia by a single surgeon (J.C.) with the aim to completely excise the tumour with a defined margin. During the procedure, the renal artery was reversibly clamped to induce period of ischemia to prevent excessive blood loss. The defect created was then closed with sutures under direct vision. The kidney was then examined for good perfusion and no obvious bleeding. The specimen is then removed and sent as a whole for histopathological examination.

# 2. Supplementary Figure 1 – Patient selection process

Number of patients excluded with reasons

(n= 565)

Radical Nephrectomies (n= 419)

Simple Nephrectomies (n=36)

T2 or above tumours (n=3)

Patients with multiple tumours (n= 45)

Patients with inherited RCC Syndromes (n=12)

Patients presented with recurrent tumour or history of PN, CRYO or RFA in the same kidney (n=50)

Patients with solitary kidney (n=34)

Number of patients assessed for eligibility to be included in this study

(n= 861)

Total number of patients included in analysis

(n= 296)

Number of patients undergoing nephrectomy, percutaneous cryoablation or radio-frequency ablation up to 2016

(n= 861)

+)

# 3. Supplementary Results to T1b subgroups

Cancer-specific survival

A total of 57 patients were evaluated for CSS after one patient was exlcuded for unknown cause of death. (Cryoablation: 31, RFA: 13, PN: 13). A total of two RCC-related deaths were observed in the cohort; one in cryoablation group and one in RFA group. CSS is comparable amongst the three groups.

Overall survival

A total of 58 patients were evaluated for OS. (Cryoablation: 31, RFA: 13, PN: 14). A total of 28 deaths were observed during the follow-up period (Cryoablation: 17, RFA: 7, PN: 4). OS is comparable amongst the three groups.

Local-recurrence free survival

A total of 57 patients were evaluated for LRFS after one patient had a lack of follow-up (Cryoablation: 31, RFA: 13, PN: 13). Seven recurrences were observed during the follow-up period (Cryoablation: 3, RFA: 1, PN: 3). LRFS is comparable amongst the three groups.

Metastasis-free survival in T1b patients

A total of 57 patients were evaluated for LRFS after one patient had a lack of follow-up (Cryoablation: 31, RFA: 13, PN: 13). Two metastases were observed; one each in the cryoablation and the RFA group. MFS is comparable amongst the three groups.

# 4. Supplementary Table 1 - Logistic regression of post-operative complication rate

|  | Odds Ratio | 95% CI | p-value |
| --- | --- | --- | --- |
| T1a | | | |
| Cryoablation | 0.76 | 0.28-2.06 | 0.593 |
| RFA | 1.37 | 0.58-3.25 | 0.469 |
| PN | Ref. |  |  |
| T1b | | | |
| Cryoablation | 2.88 | 0.31-26.68 | 0.352 |
| RFA | 2.18 | 0.17-27.6 | 0.547 |
| PN | Ref. |  |  |

The larger the odds ratio, the larger the effect of Cryoablation/ RFA has on post-operative complication rate compared to partial nephrectomy. No modalities were found to be associated with higher or lower odds of complications.

# 5. Supplementary Table 2 - Multinomial logistic regression of severity of post-operative complications

|  | Relative Risk Ratio | 95% CI | p-value |
| --- | --- | --- | --- |
| CD Grade 1 | | | |
| Cryoablation | 1.43 | 0.38-5.31 | 0.594 |
| RFA | 1.29 | 0.35-4.78 | 0.704 |
| PN | Ref. | | |
| CD Grade 2 | | | |
| Cryoablation | 0.24 | 0.03-2.19 | 0.205 |
| RFA | 1.29 | 0.35-4.78 | 0.704 |
| PN | Ref. | | |
| CD Grade 3 | | | |
| Cryoablation | 0.48 | 0.04-5.39 | 0.549 |
| RFA | 1.29 | 0.21-7.97 | 0.785 |
| PN | Ref. | | |
| CD Grade 4 | | | |
| Cryoablation | N/E | | |
| RFA | N/E | | |
| PN | Ref. | | |

The larger the risk ratio, the larger the effect of Cryoablation/ RFA has on post-operative complication severity compared to partial nephrectomy. No modalities were found to be associated with higher or lower odds of complications.

# 6. Supplementary Table 3 - Summary of all post-operative complications

| Complication | T-stage | Cryoablation | RFA | PN | p-value |
| --- | --- | --- | --- | --- | --- |
| Grade 1 | T1a | 6/72 (8.3%)  small subcapsular haematoma (x2)  Pressure point on thigh-bear hugger (x1)  Small stable pneumothorax without intervention (x1)  Small perinephric haematoma (x1)  Lateral cutaneous paraesthesia (x2) | 6/87 (6.9%)  skin burn (x1)  pain along the right lateral side  cutaneous nerve distribution (x1)  paraesthesia (x2)  pain without intervention (x1)  self-limiting macroscopic haematuria (x1) | 4/71 (5.6%)  Chest infection (x1)  Wound infection (x1)  Ileus (x1)  Other (x1) | P=0.817 |
|  | T1b | 2  reactive effusion and left lower lobe consolidation (x1)  moderate subcapsular haematoma (x1) | 1  Skin burn (x1) | 1  Wound infection (x1) | P=0.983 |
| Grade 2 | T1a | 1  Obstruction due to clots – managed conservatively (x1) | 6  small subcapsular haematoma (x3)  dropped Hb and BP - no cause found (x1)  urinary retention requiring catheter (x1)  tiny left pneumothorax  small (x1) | 4  Bleeding/ haemorrhage (x2)  Chest infection (x1)  Other (x1) | P=0.248 |
|  | T1b | 2  syncope/vasovagal episode (x1)  atrial flutter post procedure (x1) | None | None | P=0.419 |
| Grade 3 | T1a | 1  Urinary retention (x1) | 3  Ureteric stricture (x3) | 2  Bleeding/ Haemorrhage (x1)  Other (x1) | P=0.713 |
|  | T1b | 2  Urinary retention (x2) | 1  calyceal-cutaneous fistula (x1) | None | P=0.618 |
| Grade 4 | T1a | None | 1  Myocardial Infarction after general anaesthesia (x1) | None | P=0.438 |
|  | T1b | None | None | None | N/A |

# 7. Results from attempted propensity score matching analysis

The chances of receiving the different treatments were related to the matching factors. Patients receiving cryoablation or RFA tended to be much older, to have larger lesions, and to have worse Charlson indices (Table 1). In contrast, they tended to have lower grades. It was impossible to match effectively on these factors (see section 9.1 below), since after best possible matching, there were still large differences between treatment groups, particularly for age (see section 9.1 below). The mean ages were 70.5 and 57.5 years despite matching in RFA and PN groups, respectively (t=8.0, p<0.0001). Similarly, the mean ages were 68.9 and 57.8 years despite matching in cryoablation and PN group, respectively (t=5.84, p<0.001). To a lesser extent grade, lesion size, RCC type and Charlson indices differences remained between the treatment groups.9.3. Propensity Score matching histogram by treatment group showing vast differences in propensity scores by treatment (Supplementary Figure 2 and 3).

### Supplementary Figure 2 – Propensity score matching histogram of T1a RFA and PN patients

(off support indicates patients for whom matches could not be found in the matching analysis)

### Supplementary Figure 3 – Propensity score matching histogram of T1a Cryoablation and PN patients

### Supplementary Figure 4 - Distribution of age amongst partial nephrectomy, cryoablation and RFA; LPN: Partial Nephrectomy; RFA: Radio-frequency ablation; CRYO: Cryoablation

###
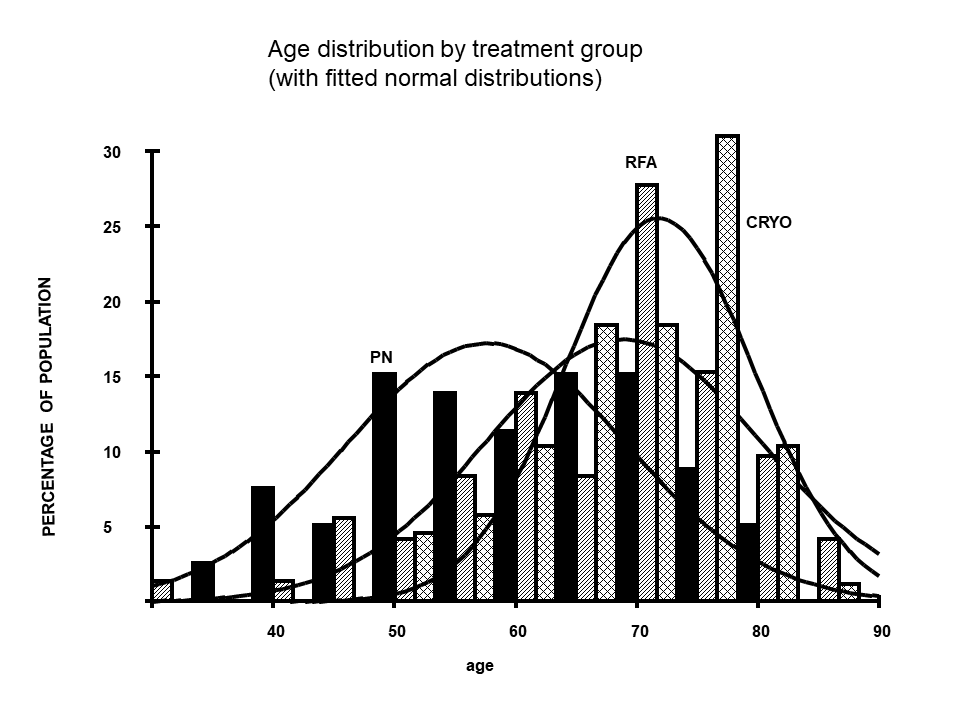


# 8. Results of multivariate analysis

### Supplementary Figure 5 – Forest plot showing magnitude of effect in T1a patients undergoing cryoablation and RFA compared to PN.

### Supplementary Table 4 – Multivariate proportional hazards regression of local recurrence-free survival in T1a cryoablation or PN patients showing 90% confidence intervals for the estimated Hazards ratios.

Cox regression -- no ties

No. of subjects = 146 Number of obs = 146

No. of failures = 5

Time at risk = 11343.5

LR chi2(10) = 20.98

Log likelihood = -11.508063 Prob > chi2 = 0.0212

------------------------------------------------------------------------------------------

_t | Haz. Ratio Std. Err. z P>|z| [90% Conf. Interval]

-------------------------+----------------------------------------------------------------

cryoablationorrfaorpn | .0026945 .0093287 -1.71 0.087 9.06e-06 .8011216

age | 1.262009 .1759648 1.67 0.095 1.003365 1.587326

|

sexnum |

Male | 14.62126 30.05952 1.30 0.192 .4970122 430.1329

|

lateralitynum |

Midline Horshoe kidney | 2.37e-21 . . . . .

Right | .6337033 .8054968 -0.36 0.720 .0783211 5.127355

|

charlson2 | 1.56277 .9100751 0.77 0.443 .5996456 4.072822

1.charlson3 | 3.15e+17 . . . . .

preegfr | 1.019959 .0325701 0.62 0.536 .9677688 1.074964

|

grade |

1 | 8.72e-09 . . . . .

2 | 4.39e+10 6.61e+10 16.28 0.000 3.69e+09 5.22e+11

3 | 3.72e+10 . . . . .

4 | 1.53e-08 . . . . .

|

renal2 | 3.143127 1.733327 2.08 0.038 1.268884 7.785777

lesionsizecm | 4.025748 5.130869 1.09 0.275 .4947626 32.7564

|

rcctypeconventional1papi |

2 | 2.84e-20 . . . . .

3 | 6.02e-09 . . . . .

4 | .4670436 .8128928 -0.44 0.662 .0266701 8.17881
